# The large extracellular loop of CD63 interacts with gp41 of HIV-1 and is essential for establishing the virological synapse

**DOI:** 10.1038/s41598-021-89523-7

**Published:** 2021-05-11

**Authors:** Daniel Ivanusic, Kazimierz Madela, Norbert Bannert, Joachim Denner

**Affiliations:** 1 HIV and Other Retroviruses, Robert Koch Institute, Nordufer 20, 13353 Berlin, Germany; 2 Special Light and Electron Microscopy, Robert Koch Institute, Nordufer 20, 13353 Berlin, Germany; 3 Institute of Virology, Department of Veterinary Medicine, Free University Berlin, 14163 Berlin, Germany

**Keywords:** Cell biology, Immunology

## Abstract

Human immunodeficiency virus type 1 (HIV-1) persists lifelong in infected individuals and has evolved unique strategies in order to evade the immune system. One of these strategies is the direct cell-to-cell spread of HIV-1. The formation of a virological synapse (VS) between donor and target cell is important for this process. Tetraspanins are cellular proteins that are actively involved in the formation of a VS. However, the molecular mechanisms of recruiting host proteins for the cell–cell transfer of particles to the VS remains unclear. Our study has mapped the binding site for the transmembrane envelope protein gp41 of HIV-1 within the large extracellular loop (LEL) of CD63 and showed that this interaction occurs predominantly at the VS between T cells where viral particles are transferred. Mutations within the highly conserved CCG motif of the tetraspanin superfamily abrogated recruiting of expressed HIV-1 GFP fused Gag core protein and CD63 to the VS. This demonstrates the biological significance of CD63 for enhanced formation of a VS. Since cell–cell spread of HIV-1 is a major route of persistent infection, these results highlight the central role of CD63 as a member of the tetraspanin superfamily during HIV-1 infection and pathogenesis.

## Introduction

Human immunodeficiency virus type 1 (HIV-1), as a retrovirus, has evolved several strategies in order to avoid clearance by the adaptive immune system. Establishment of a sustainable infection in the host is the most challenging and significant step for HIV-1. HIV-1 can infect CD4+ cells by free floating virions and through cell-to-cell contact. This cell-to-cell transfer is considered as a predominant spread of HIV-1 and leads to an extremely efficient infection route, which is up to 18,000-fold more efficient than infection carried out by cell-free particles^1–4^. Especially in lymph nodes, where cells are densely packed, HIV-1 can be efficiently relocated from infected to uninfected cells^5^. Direct virus transmission occurs by a preceding formation of a cell-to-cell membrane site that connects pre- and postsynaptic cells termed as virological synapse (VS). The VS is formed by the interaction of transmembrane proteins of both cells that tightly surround a small space separated from the extracellular environment (see for review^6^). HIV-1 contains only 15 proteins and RNA^7^, and it is obvious that HIV-1 has to utilize host proteins and is involved in multiple protein–protein interactions during infection and replication steps^8,9^. According to the current model, the spread of HIV through a VS is driven by an interaction of Env from the infected cell and CD4 of the non-infected cell, and transmission is triggered by an interaction between intercellular adhesion molecule 1 (ICAM-1) and lymphocyte function-associated antigen 1 (LFA-1)^10–13^. The core protein of HIV-1, Gag, is recruited to the VS after the formation of Env-dependent stable cell-to-cell adhesions^14^. There are reports showing that small integral type III membrane proteins belonging to the tetraspanin family are involved in the formation of the VS and regulate cell-to-cell transfer of HIV^15–17^. The main characteristics of tetraspanins are 4 transmembrane (4TM) domains, a small extracellular loop (SEL), a large extracellular loop (LEL) and the highly conserved CCG motif in the LEL. This motif and other cysteines in the LEL are important for the formation of two to four disulfide bridges that are required for correct folding and conformation of the LEL^18,19^. Tetraspanins are highly organized in tetraspanin enriched microdomains (TEMs) on the cell surface, where tetraspanins are associated with other tetraspanins or membrane proteins, such as integrins or receptors^20,21^. These TEMs act as physical interacting platform units concentrating interaction partners within TEMs^22^. The fact that tetraspanins are exposed < 5 nm above the plasma membrane makes them an ideal docking station for other transmembrane proteins^23^. How CD63, one of these tetraspanins, and other transmembrane proteins are recruited to the VS and the role they play in the subsequent cell-to-cell transfer of HIV particles is not well understood^24^. There are several lines of evidence supporting a role of tetraspanins in the replication process of HIV, mainly in very early or late events. In this respect, it has been demonstrated that HIV-1 particles contain selectively incorporated CD63 as well as other tetraspanins^25–27^. Furthermore, this incorporation negatively regulates the infectivity of HIV particles^28^. CD63 and other tetraspanins, such as CD81, can be found at VS sites, and it has been shown that tetraspanins regulate cell–cell transfer of HIV-1^15^. In this report, we provide evidence that CD63 interacts with the transmembrane envelope protein gp41 of HIV-1 in the context of a VS. Moreover, our data identify the LEL of CD63 as the major interaction domain with the viral gp41 protein, highlighting the role of this tetraspanin during cell-to-cell transfer of HIV-1 particles. The findings provide novel insights concerning the mechanism how CD63 might act as a regulatory protein for cell-to-cell transfer.

## Results

### Mapping of the CD63 interaction domain

**Figure 1:**
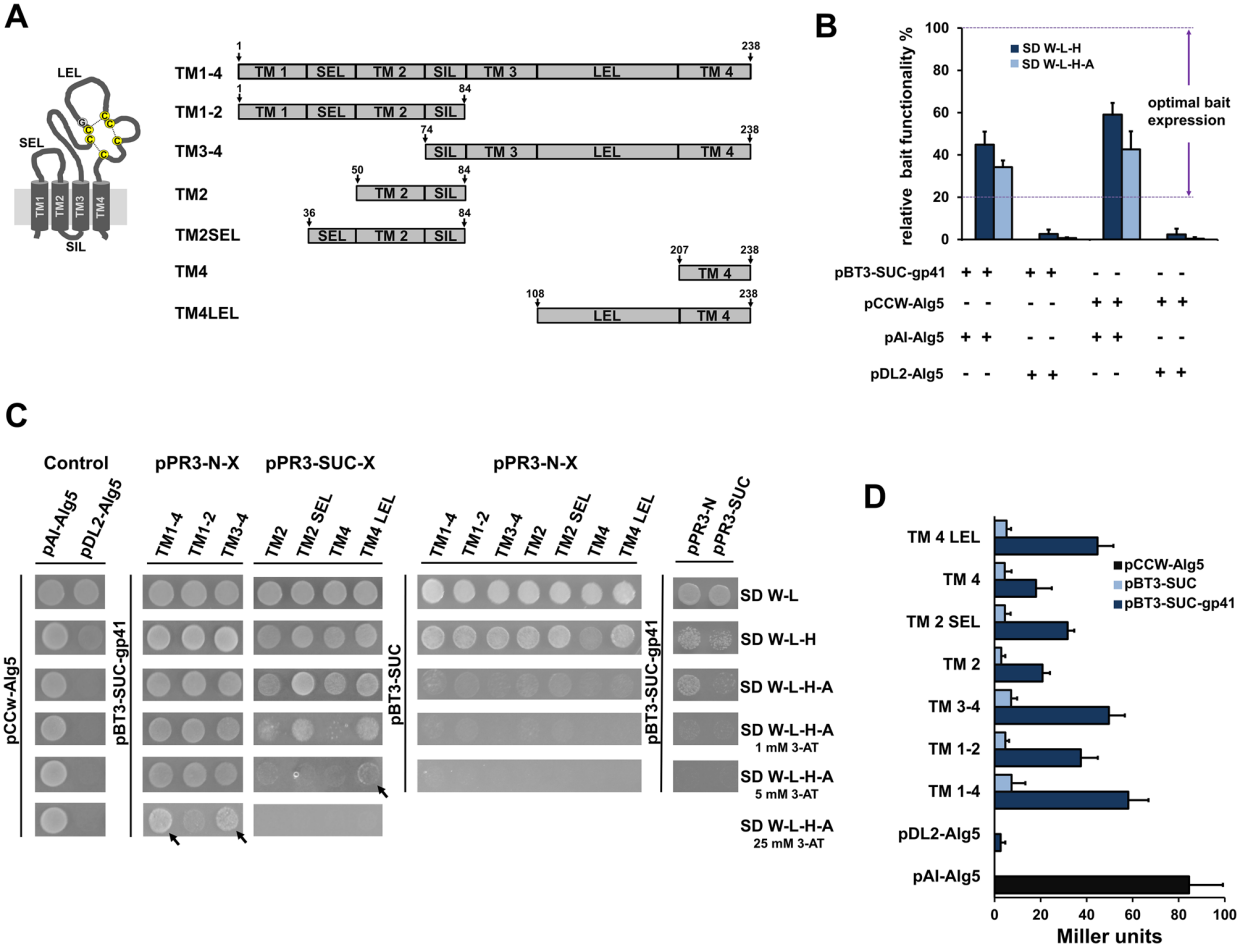
Mapping of the CD63 domain involved in the interaction with gp41. (**A**) Schematic representation (not to scale) of CD63 domains used for interaction mapping, numbers refer to the amino acid sequence. TM, transmembrane domain; SIL, small intracellular loop; SEL, small extracellular loop; LEL, large extracellular loop; C, cysteine; G, glycine; disulfide bridges are illustrated by broken lines. (**B**) Control assay to ensure functionality of the bait construct. NMY51 yeast cells were transformed with the pBT3-SUC-gp41 or pCCW-AIg5 control bait vector along with positive pAI-Alg5 (+) and negative pDL2-Alg5 (−) prey control vectors and then grown on SD W-L–H or SD W-L-H-A selective plates. (**C**) NMY51 interaction yeast growth assay. Yeast cultures transformed with the indicated bait and prey vectors were plated on plates containing synthetic dropout (SD) media without the indicated components (W, L, H, and A) and with 3-aminotriazole (3-AT) supplementation at different concentrations. Strongest yeast growth within 3-AT gradient is marked with an arrow. (**D**) Quantification of β-galactosidase activity. Each value is given in Miller units and represents the result of β-galactosidase activity assays using three independent yeast colonies.

Using a membrane-based split ubiquitin screen, we have identified full-length CD63 from a Jurkat T cell cDNA library as a binding partner of gp41^29^. This yeast-based screening method is suitable to detect novel interactions between membrane proteins in their native conformation in vivo^30,31^. To analyze the interaction in more detail and to reveal the CD63 domains involved, pair-wise interaction testing of CD63 domains (Fig. 1A) with gp41 were performed. As bait for these experiments, a previously described vector named pBT3-SUC-gp41 was used^29^. It consists of a cDNA fragment encoding the ectodomain and transmembrane region of gp41 (HIV-1 Env aa 534–709) cloned into the pBT3-SUC vector upstream in-frame with the C-terminal part of yeast ubiquitin (C_ub_) and the LexA-VP16 reporter cassette. We used the prey vector pPR3-N for cloning CD63 variants containing two transmembrane domains and pPR3-SUC for single pass membrane variants. In an initial experiment using positive and negative control prey-vectors, the general functionality of the bait vector was confirmed, as well as the membrane localization of the expressed bait in a cis position by using pCCW-AIg5 bait reference control (Fig. 1B). Co-expression of the bait along with the control prey Alg5-N_ub_I resulted in a reconstitution of split-ubiquitin through the strong affinity of wild-type N-terminal part of yeast ubiquitin (N_ub_I) for C_ub_ and the concurrent activation of reporter genes. In contrast, when the bait is co-expressed together with the negative control prey pDL2-Alg5 reporter genes were not activated because the mutated N_ub_G (I13G) moiety had no affinity for C_ub_^29,30,32^. After generating a different set of CD63 mutant prey vectors, experiments were performed to analyze the interaction of CD63 variants with the bait pBT3-SUC-gp41 in order to identify the CD63 interacting domain. The interaction analysis was monitored by yeast growth and quantification of β-galactosidase. Transformed yeast colonies containing the full-length CD63 sequence TM1-4 (aa 1–238), sequence TM 3–4 (aa 74–238) and single membrane spanning mutant variant containing the TM4LEL (aa 108–238) showed a strong growth on selective plates SD W-L-H-A supplemented with 3-Amino-1,2,4-triazole (3-AT) (Fig. 1C). 3-AT competitively inhibits the *HIS3* gene product, while 3-AT supplementation reduces the background growth (leakiness of the *HIS3* reporter) due to *HIS3* gene activity in the absence of a protein–protein interaction (PPI)^33,34^. Autoactivation of each construct was tested against prey or bait empty vectors, and background yeast growth was inhibited at a 3-AT concentration of 1 mM (Fig. 1C). A quantitative determination of the β-galactosidase activity confirmed that the strongest interaction took place with CD63 derived prey variants containing the LEL (Fig. 1D). These results indicate that the LEL residues of CD63 are important for the interaction with gp41.

### Point mutations introduced in the CCG motif of CD63 abrogate the interaction with gp41

**Figure 2:**
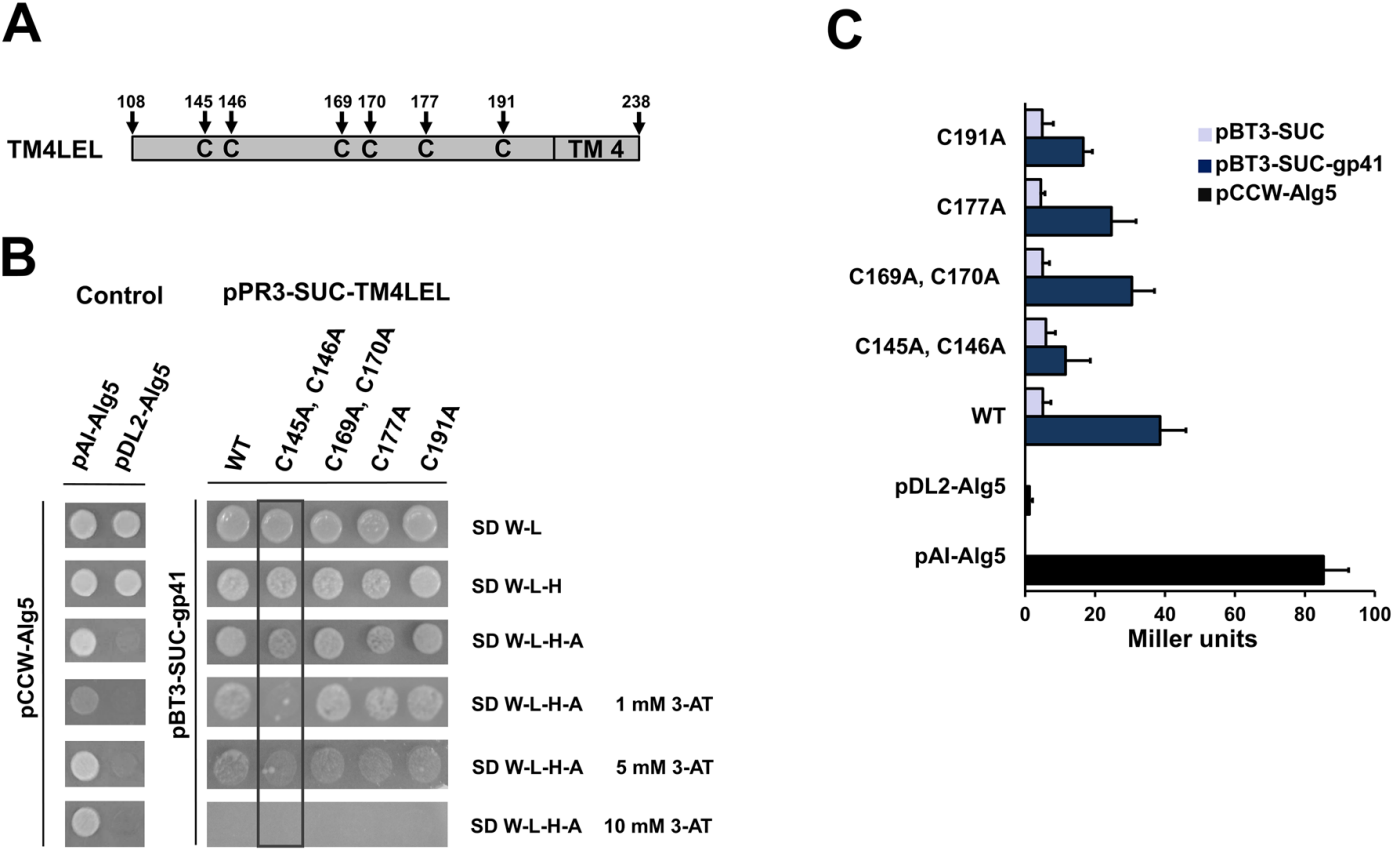
Impact of cysteine mutations in the LEL sequence of CD63 on the interaction with gp41. (**A**) Schematic representation of the cysteine positions within the expression prey construct pPR3-SUC-TM4LEL (not to scale). TM4LEL, the transmembrane domain 4 and LEL of CD63 (**B**) NMY51 interaction yeast growth assay**.** Yeast cultures co-transformed with the pBT34-SUC-gp41 bait and prey vector pPR3-SUC-TM4LEL containing the indicated C→A point mutations were plated on SD selective plates. The weakest yeast growth is marked with a frame. (**C**) β-galactosidase activity was assayed for each co-transformed yeast culture in order to address the interaction strength. Each value is given in Miller units and 5 represents the result of β-galactosidase activity assay using three independent yeast colonies.

The LEL of CD63 contains six cysteines^35^. In order to analyze the significance of cysteine loops for the interaction with gp41, we generated cysteine mutations (C→A) in the CD63LEL of the prey plasmid pPR3-SUC-TM4LEL (Fig. 2A). Co-transformation of the bait pBT3-SUC-gp41 and prey pPR3-SUC-TM4LEL containing the wild-type CD63LEL sequence revealed the strongest yeast growth on selective SD W-L-H-A plate supplemented with 3-AT 5 mM when comparable with CD63LEL containing generated cysteine mutation sites C169A, C170A, C170A and C191A (Fig. 2B). However, the LEL with cysteine mutation sites C145A and C146A showed a strong defect in yeast growth on selective SD W-L-H-A plates supplemented with 3-AT 1 mM (Fig. 2B). At this concentration of 3-AT, the autoactivity from control yeast transformations with the prey TM4LEL showed no background growth (Fig. 1B). The quantification of the β-galactosidase activity confirmed that the interaction with cysteine mutations at positions C145A and C146A within the evolutionary highly-conserved tetraspanin CCG motif is very weak compared to other mutation sites (Fig. 2C). In contrast, the introduced cysteine point mutation sites C169A and C170A in the LEL showed a beta-galactosidase activity that was comparable to the wild-type LEL sequence. Interestingly, the point mutations site C191A lead to a reduced β-galactosidase activity, but no reduced yeast growth was observed. This observation can be explained by the failed formation of a disulfide bridge^36^ of the first cysteine at aa position 145 from the CCG motif with the cysteine at aa position 191 (Fig. 1A). These results indicate that the LEL of CD63 formed by the first cysteine in the LEL within the CCG motif is important for the interaction with gp41 and sufficient for confirmation of the specific PPI found in the split-ubiquitin assay.

### Fluorescence resonance energy transfer (FRET) analysis confirmed the pivotal role of the CCG motif within the LEL of CD63 in the interaction with gp41

**Figure 3:**
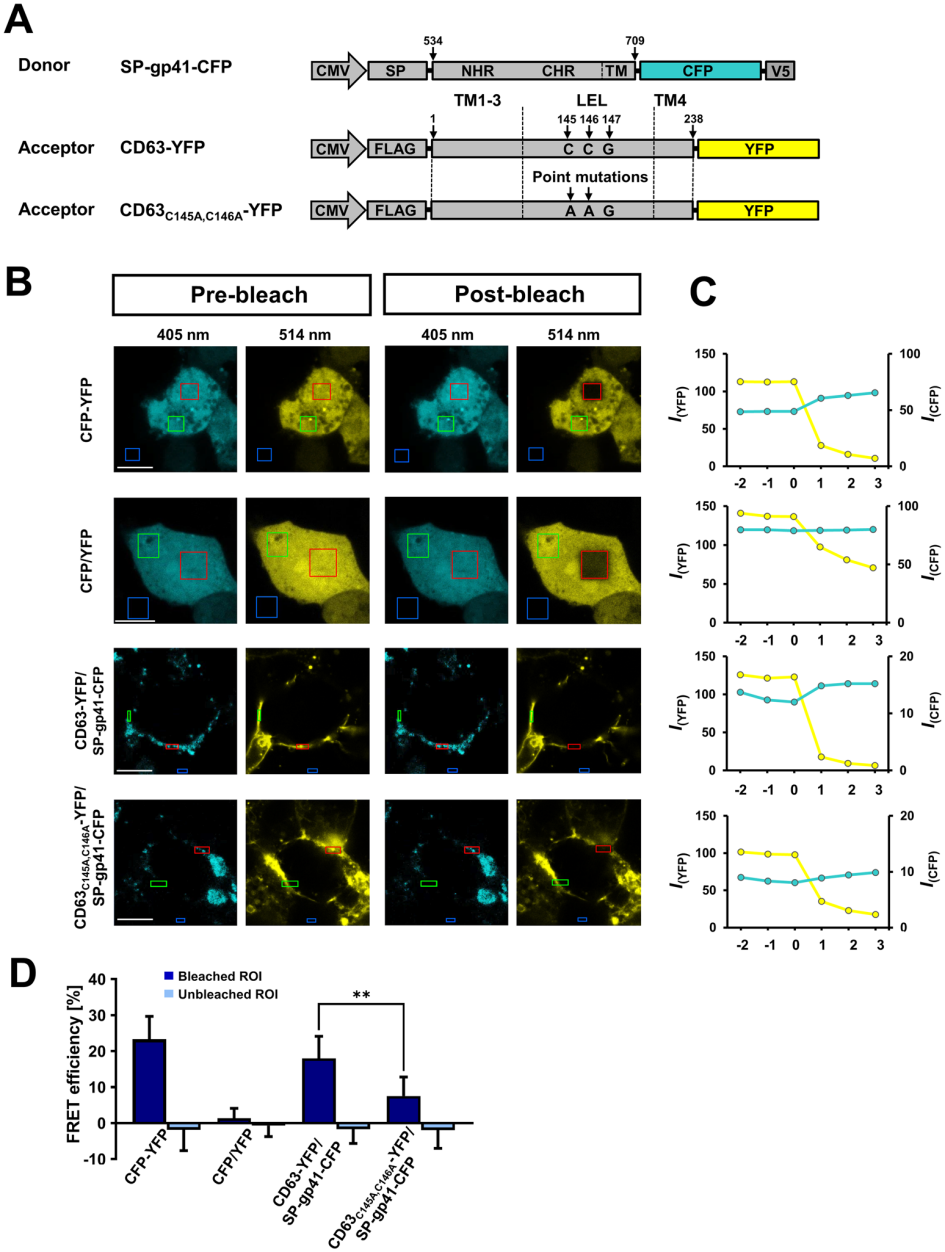
Detection of direct interactions between CD63 and gp41 by FRET. (**A**) Schematic representation of vectors used for the FRET experiments (not to scale), CMV: cytomegalovirus promoter, SP: sequence of murine Ig kappa-chain V-J2-C, NHR: N-terminal heptad repeat of gp41, CHR: C-terminal heptad repeat of gp41, CFP: cyan fluorescent protein, V5 tag: GKPIPNPLLGLDST, CD63: cluster of differentiation 63, YFP: yellow fluorescent protein, FLAG tag: DYKDDDDK, numbers refer to the amino acid sequence. (**B**) Confocal images of HEK293T cells expressing FRET pairs. Areas are marked by frames as regions of interest (ROIs) in red (bleached), green (unbleached) and blue (background) frames. (**C**) Fluorescence intensity (*I*) profiles of CFP (cyan line) and YFP (yellow line) emissions during acceptor photobleaching experiments. Acceptor photobleaching with a 514 nm laser line starts at time point 0 and is repeated at each further time point. (**D**) Quantification of FRET acceptor photobleaching experiments by calculating FRET efficiencies for CFP-YFP (n = 6), CFP/YFP (n = 6), CD63-YFP/SP-gp41-CFP (n = 9), and CD63_C145A,C146A_-YFP/SP-gp41-CFP (n = 9). All scale bars: 10 µm.

Yeast-based interaction data have to be confirmed by alternative methods in order to exclude false positive interaction partners. In a first approach, a FRET method was used and acceptor photobleaching experiments were performed. HEK293T cells were transfected with plasmids encoding cyan fluorescent protein (CFP) as the energy donor and yellow fluorescent protein (YFP) as the acceptor fused to gp41 or CD63 sequences, respectively (Fig. 3A). This approach involves monitoring of the donor (SP-gp41-CFP) fluorescence signals in the presence (pre-bleach) and absence (post-bleach) of the acceptor (CD63-YFP). The donor construct contains a signal sequence (SP) from Ig kappa-chain V-J2-C^37^. The fluorescence intensity profiles of the FRET bleaching experiments were examined 24 h post-transfection. We observed that, for the FRET pair SP-gp41-CFP/CD63-YFP, the CFP fluorescence intensity increased after acceptor photobleaching comparable to the positive control CFP-YFP at several time points (Fig. 3B,C). Calculated FRET efficiencies obtained from acceptor photobleaching experiments are summarized in Fig. 3D. These data show that the FRET efficiency calculated for the interaction pair SP-gp41-CFP/CD63-YFP can be compared to the positive control where CFP and YFP are expressed as a single fusion protein. This observation revealed that CD63-YFP/SP-gp41-CFP contained a bimolecular interaction characteristic. In contrast, the expressed FRET pair SP-gp41-CFP/CD63_C145A,C146A_-YFP showed up to 58% reduced FRET efficiency values after acceptor photobleaching of YFP compared to when used CD63-YFP as acceptor. However, the FRET efficiency calculated for the expressed FRET pair SP-gp41-CFP/CD63_C145A,C146A_-YFP were different from the pseudo-FRET efficiencies obtained from separately expressed CFP and YFP (Fig. 3D). In summary, these data confirm that CD63 and gp41 interact directly, and the CCG motif is important for this PPI.

### Proximity ligation assays (PLAs) confirmed the interaction between CD63 and gp41

**Figure 4:**
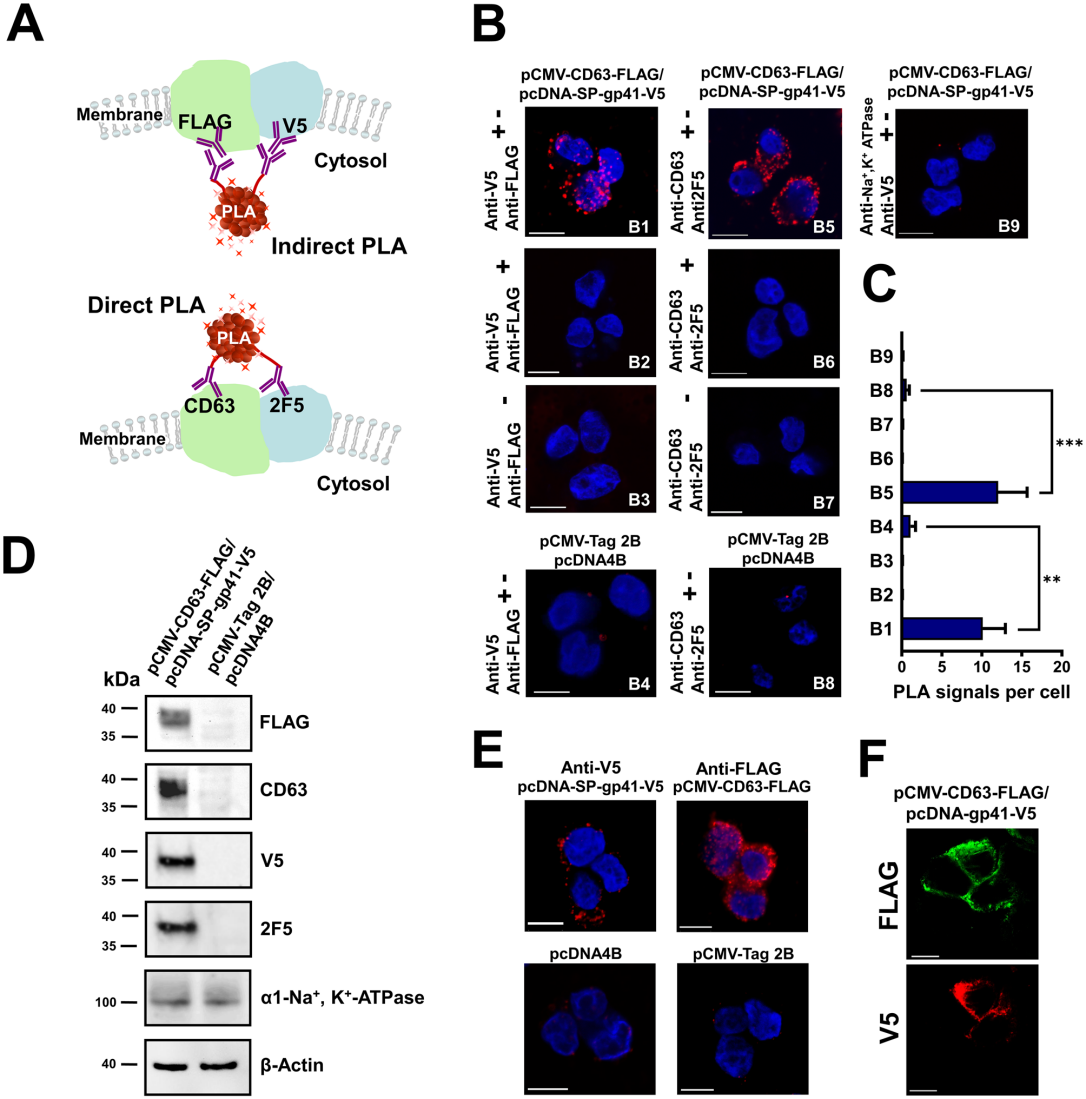
Detection of the interaction between CD63 and gp41 by PLA. (**A**) A schematic illustration of direct and indirect PLA experiments. Indirect PLA was performed by detecting FLAG and V5 epitopes by primary antibodies and direct PLA by conjugation of primary antibodies recognizing CD63 and gp41 (2F5) specific epitopes. (**B**) Confocal images of HEK293T cells co-transfected with indicated vectors. Indirect PLA was performed on B1–B4, B9 and direct PLA on B5–B8. (**C**) Quantification of PLA signals per cell obtained for the expressed protein pair SP-gp41-V5/CD63-FLAG from independent transfections: B1 (n = 100), B2 (n = 97), B3 (n = 100), B4 (n = 100), B5 (n = 110), B6 (n = 96), B7 (n = 95), B8 (n = 105), and B9 (n = 160). (**D**) Western blot analysis of transfected HEK293T cells used for PLA experiments expressing CD63-FLAG and SP-gp41-V5; whole-cell lysates were probed with the indicated primary antibodies. (**E**) Confocal images of protein expression control by FLAG and V5 by single recognition PLA. (**F**) Confocal images of indirect immunofluorescent staining for expressed CD63-FLAG and SP-gp41-V5 using transfected HEK293T cells from PLA experiments.

To evaluate the PPI found between CD63 and gp41 conditions without fluorescently labeled proteins, we performed quantitative in situ PLAs on HEK293T. PLA experiments require a pair of primary antibodies raised in different species, which is used to recognize the PPI of interest. The detection of bright red PLA dot signals is only possible when both interacting proteins are in close proximity and the conjugated DNA with protein specific antibodies can form a DNA circle. This circle is then ligated and hybridized with probes^38,39^. Expression vectors were used to generate CD63 fused with FLAG (pCMV-CD63-FLAG) and gp41 fused with V5 (pcDNA-SP-gp41-V5) antibody-binding epitopes. Pair-wise interaction PLA tests between the expressed CD63-FLAG and SP-gp41-V5 proteins were performed in HEK293T cells, and primary antibodies against the fused tag and against an epitope within the expressed proteins were used. First, PLA experiments were performed employing indirect PLA (Fig. 4A, images B1–B4) using primary antibodies against FLAG and V5 epitopes in combination with Duolink secondary antibodies conjugated with Duolink DNA oligonucleotides mouse PLUS (+) and goat MINUS (−). Second, using direct PLA, CD63 and gp41 (2F5) epitope binding antibodies were conjugated with PLA Duolink DNA oligonucleotides PLUS (+) and MINUS (−) (Fig. 4A, images B5–B8). Transfected cells expressing CD63-FLAG and SP-gp41-V5 showed several bright PLA signals. In contrast, in controls, only a few weak and scattered PLA signals were observed. In order to demonstrate the precision of the assay, cellular Na^+^, K^+^-ATPase was used as a reference protein^40^. This indirect PLA control experiment showed an absence of specific PLA signals, which means that SP-gp41-V5 is not in close proximity with the Na^+^, K^+^-ATPase. This confirmed that the PLA experiments performed specifically detected an interaction between two proteins in a pool of expressed proteins in the same compartment (Fig. 4B9). Quantification revealed at least a tenfold difference when cells were transfected with pCMV-CD63-FLAG/pcDNA-SP-gp41-V5 in the number of PLA signals per cell (Fig. 4C). Western blot analysis confirmed protein expression driven by transfected vectors, as well endogenous protein expression in HEK293T cells used for PLA experiments (Fig. 4D). Further, when single recognition PLA using anti-V5 or anti-FLAG polyclonal goat antibodies were performed, several PLA signals were detected on the surface of HEK293T cells, confirming localization of expressed CD63-FLAG and SP-gp41-V5 on the cell membrane (Fig. 4E). This PLA variant uses only one polyclonal primary antibody in order to detect close proximity of different epitopes within one protein species. In addition, applying an indirect immunostaining technique, it was shown that expressed CD63-FLAG and SP-gp41-V5 were predominantly expressed in the plasma membrane (Fig. 4F). These results corroborate and extend our previous finding indicating expression of CD63-FLAG and SP-gp41-V5 on the surface where both proteins are in close proximity, qualifying for a PPI.

### Proximity of CD63 and gp41 at the VS

**Figure 5:**
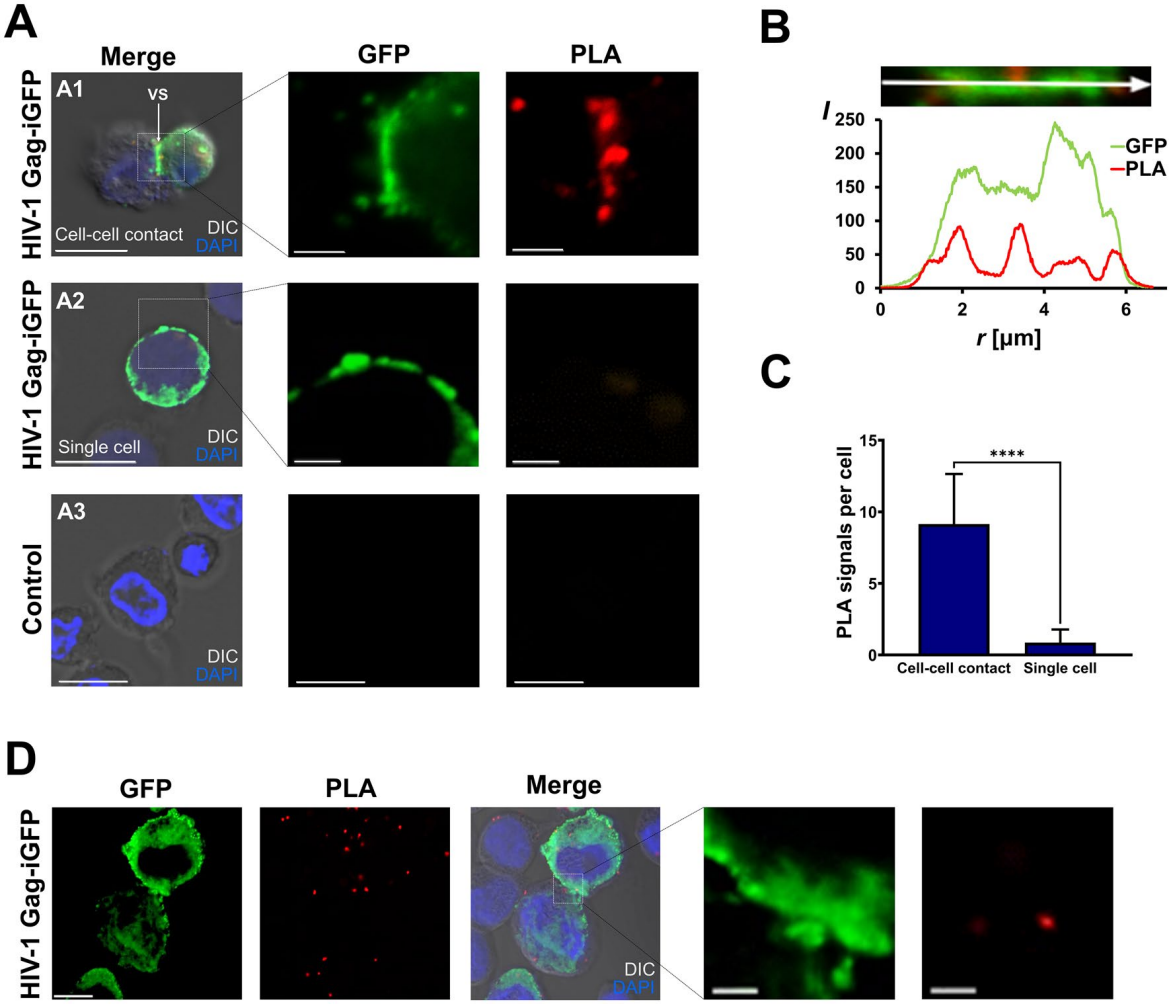
(**A**) Detection of interaction between CD63 and gp41 at the virological synapse (VS) between Jurkat cells expressing HIV-1_JR-FL_Gag-iGFP by employing direct PLA against CD63 and 2F5 epitopes. The control contains Jurkat cells without HIV-1_JR-FL_Gag-iGFP transfection. (**B**) Plot of fluorescence intensities for GFP and PLA signals along the VS (white arrow). (**C**) Quantification of PLA signals obtained for Jurkat cells with cell–cell contacts (n = 14) and for single cells without cell–cell contacts (n = 15). (D) Direct PLA against CD63 and 2F5 using HEK293T cells. All scale bars are 10 µm, except in cutouts, where they are 2 µm.

To explore where the PPI between CD63 and gp41 described above is formed under physiological relevant conditions, we attempted to answer this question with direct PLA experiments using Jurkat cells expressing a molecular clone encoding HIV_JR-FL_ Gag-iGFP. This approach enables to probe the found interaction between CD63 and full-length gp41 in the context with all expressed HIV-1 proteins. Especially the expression of gp120 that undergo a trimeric association with gp41 (pre-fusion confirmation) will enable to reach close physiological conditions. This HIV-1 clone is based on the NL4-3 isolate but contains the R5-tropic envelope of the JR-FL isolate in addition to a GFP gene inserted between the matrix and the capsid sequence flanked by the viral protease cleavage sites SQNYPIVQ^41^. The HIV JR-FL envelope was selected in order to avoid infection by free floating virus particles of Jurkat cells. It was shown that cell-to-cell transfer is independently from CCR5 co-receptor expression^2^. At 24 h post-transfection with HIV_JR-FL_ Gag-iGFP, the Jurkat cells were prepared for PLA experiments. PLA signals were observed particularly at intercellular cell–cell contact sites consistent with VS structures but not on other cell sites (Fig. 5A1). At these contact sites, GFP signals were abundant, indicating significant formation of a VS for viral transfer. In contrast, in the absence of cell-to-cell contacts, PLA signals were rarely detected (Fig. 5A2). In parallel experiments with Jurkat cells that were not transfected with the molecular HIV_JR-FL_ Gag-iGFP clone, PLA signals were not observed (Fig. 5A3). The fluorescence intensity for PLA signal along the VS showing that PLA signal appeared with increased GFP intensity (Fig. 5B). Quantification of PLA signals indicated high levels of PLA signals when a VS was established between Jurkat cells (Fig. 5C). When HEK293T cells were used we did not observe comparable amount of PLA signals at cell–cell contacts where GFP signals are polarized (Fig. 5D). PLA signal distribution having similar characteristics with Jurkat cells that are not in contact with other cells where GFP signals are not polarized. Taken together, these results suggest that CD63 interacts with the gp41 at cellular contact sites of CD4+ cells enriched with HIV Gag-iGFP signals indicating the virological synapse.

### The CCG motif within the LEL of CD63 is essential for establishing of a VS and recruiting HIV-1_JR-FL_Gag-iGFP formations to the VS

**Figure 6:**
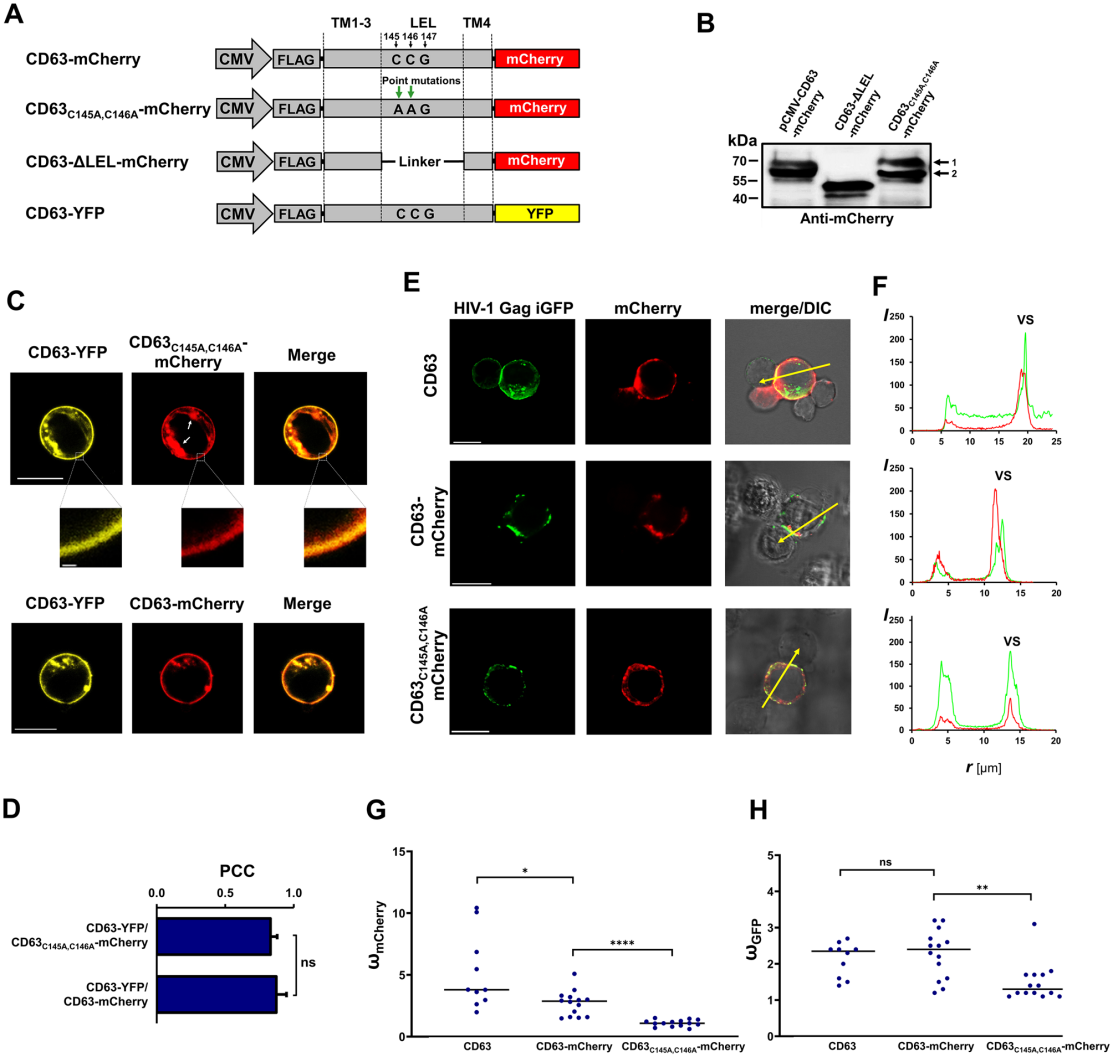
Influence of cysteine mutations within the LEL on the CD63 and HIV-1_JR-FL_Gag-iGFP recruitment to the VS. (**A**) A schematic representation (not to scale) of the fused mCherry or YFP CD63 expression constructs, numbers refer to the amino acid sequence. (**B**) Western blot analysis of transfected HEK293T cells with constructed vector. Molecular weight of glycosylated CD63 (1) and CD63 backbone (2) is marked with a black arrow. (**C**) Colocalization of expressed CD63 variants, arrows point to observed higher intracellular protein localization, cut-out shows colocalization details at the plasma membrane. (**D**) Calculated Pearson coefficients of correlation (PCC) for n = 11 cells are graphed. (**E**) Confocal images of Jurkat cells transfected with HIV-1_JR-FL_Gag-iGFP and (second and third row) with pCMV-CD63-mCherry or pcmv-CD63_C145A C146A_-mCherry. Jurkat cells were indirectly stained with anti-CD63 antibodies (first row) in order to visualize endogenous CD63 expression. The yellow arrow indicates the region for which the fluorescence intensity (*I*) was measured. (**F**) The*I*values for GFP and mCherry are displayed as a function of the distance. Quantification of recruiting (**G**) CD63 and (**H**) Gag proteins to the VS by calculation of the recruiting factor ω was dependent on mCherry or GFP obtained from n = 14 (CD63-mCherry-FLAG, CD63C_145A,C146A_-mCherry-FLAG) and n = 10 (CD63 independent cell–cell contacts from Jurkat cells measured from three positions within each cell, mean values are displayed as dots. All scale bars are 10 µm, except in (**C**), where they are 0.5 µm.

CD63 expression vectors fused C-terminally with the fluorescent protein mCherry or YFP, as well as a CD63 version tagged with mCherry containing C145A,C146A mutations in the LEL (Fig. 6A), were generated in order to analyze colocalization and to monitor the dynamics of these proteins at the VS by confocal microscopy. The Western blot analysis of cell lysates showed that the expression levels of the wild-type and mutant CD63-mCherry proteins were similar and that generated expression vectors are functional (Fig. 6B). CD63-mCherry versions containing the LEL show higher molecular weight (70 kDa) than the backbone CD63-mCherry (55 kDa) and is indicative for glycosylation of the LEL^42^. CD63 construct version with a deleted LEL was used in this case to show the expression without the glycosylated LEL. Difference of the protein location between CD63-mCherry and CD63_C145A,C146A_-mCherry at the plasma membrane was not observed (Fig. 6C). Confocal images suggest that the intracellular localization of CD63_C145A,C146A_-mCherry is slightly higher than of CD63-YFP or CD63-mCherry. However, the calculation of the Pearson correlation coefficient (PCC) indicates high degree of correlation between fluorescence signals emitted from CD63-YFP/CD63_C145A,C146A_-mCherry (mean R = 0.83) and are comparable to the correlation between CD63-YFP/CD6-mCherry (mean R = 0.87) that was used as a wildtype reference (Fig. 6D). These observations indicate that the constructed vector expressing CD63_C145A,C146A_-mCherry is an applicable control allowing to study the importance of CD63LEL at the VS. We transfected therefore Jurkat cells with the molecular clone HIV-1_JR-FL_Gag-iGFP and pCMV-CD63-mCherry or pCMV-CD63_C145A,C146A_-mCherry. In fixed samples, several cell–cell contacts were analyzed by confocal microscopy and revealed that fluorescence signals emitted from Gag-iGFP, endogenous CD63 and wild-type CD63 fused with mCherry are mainly detectable at the VS between infected and uninfected Jurkat cells. In contrast, CD63 with introduced cysteine mutations C145A, C146A showed a randomized distribution on the surface of Jurkat cells (Fig. 6E). The intensities of the fluorescence peaks for endogenous CD63 and CD63-mCherry correlated with the accumulation of HIV-1_JR-FL_Gag-iGFP formations at the VS (Fig. 6F). Interestingly, the fluorescence intensities of HIV-1_JR-FL_Gag-iGFP suggest that there is an absence of CD63_C145A,C146A_-mCherry polarization at the VS (Fig. 6E,F). Furthermore, the recruitment of CD63 protein and HIV-1_JR-FL_Gag-iGFP on several Jurkat cell–cell contacts was quantified. For this, the relative increase of the protein level in one cell–cell contact for the green (GFP) and the red channel (mCherry) recruitment factor ω was calculated by employing fluorescence intensity data. The value ω = 1 means that the fluorescence intensity of expressed proteins is evenly distributed over the cell surface, and ω > 1 means that the proteins are highly concentrated at the VS. These results indicate no significant recruitment to the VS for CD63_C145A,C146A_-mCherry and a slightly decreased recruitment of the CD63-mCherry protein in comparison to endogenous expressed CD63 protein (Fig. 6G). A further calculation of the ω value for the GFP signals showed that the expression of CD63_C145A,C146A_-mCherry significantly reduced the recruitment of HIV-1_JR-FL_Gag-iGFP to the VS (Fig. 6H).

## Discussion

Yeast-based split ubiquitin screen and pair-wise interaction analysis identified CD63 as an interaction partner of gp41^29^, and this finding was confirmed by the correlative FRET-PLA technique^40^. Our previously performed PPI analysis was appropriate to address the question of whether CD63 and gp41 can interact, but it did not provide details on where or under which circumstances the identified interaction happens. In this study, the yeast-based split ubiquitin interaction assay was utilized to map the interaction domain. We found that the LEL of CD63 is responsible for interaction with gp41. This lateral interaction position was demonstrated by testing the gp41 bait with control prey vectors where N_ub_ and C_ub_ only fused together when they are in a lateral position (Fig. 1B–D). This is consistent with reports that tetraspanins interact with other membrane proteins in a lateral position and that those interactions are mainly mediated by the LEL^43,44^. Members of the tetraspanin superfamily contain conserved cysteine residues within the LEL, among them is the CCG motif, which is highly conserved^45,46^. The logical step was to introduce C→A point mutations in the LEL in order to cause a major local change in the CD63 loop structure. Constructs containing mutations are powerful tools to specifically disrupt the interaction between proteins^47^ and testing of the altered LEL sequence showed the specificity of the employed yeast-based split ubiquitin interaction assay and provides additional evidence for positive interactions. When a prey encoding the wild-type CD63 and a set of mutated prey constructs coding for CD63TM4LEL were used, it was observed that the CCG motif was essential for the interaction with the gp41 bait. However, it is unlikely that cysteines of the CCG would directly be involved in the interaction between CD63 and gp41 through residue-residue contacts, but they are important for the structural and interaction integrity of the LEL. Due to the important role of disulfide bonds in folding and stabilizing the tertiary structure of proteins^48^, their removal most likely causes a misfolding of the LEL. Misfolded regions can be used to determine important interaction regions within a protein. For CD81, a member of the tetraspanin superfamily, it has been shown that cysteines within the LEL are important for stabilizing the protein folding^49^. The same observation was made with FRET experiments where the point mutations C145A and C146A led to a decrease in PPI strength (Fig. 3). Performing interaction experiments in HEK293T cells, a close proximity between recombinant expressed SP-gp41-V5 and CD63-FLAG proteins was detected by indirect and direct PLA (Fig. 4B,C). In addition, the PLA results demonstrate that the employed PLA technique is very specific and able to detect the interaction between CD63 and gp41 in a pool of several expressed proteins, such as the reference protein Na^+^, K^+^-ATPase^40^. Further, PLA signals were observed exclusively at cell–cell contacts between Jurkat cells expressing the molecular clone HIV_JR-FL_ Gag-iGFP (Fig. 5A). These cell contacts are termed VSs and represent multimolecular complexes at the interface between infected and uninfected cells^1,50^. At this point, our investigations were focused on the protein recruiting and dynamics at the VS, and it was shown that the wild-type CD63-mCherry was recruited to the VS together with HIV-1_JR-FL_Gag-iGFP formations. Previous analysis of the VS performed by electron microscopy showed that small GFP puncta as we detected are indicative for the presence of HIV-1 particles in the target cell^12^. The overlap of Gag-iGFP fluorescence signals correlated strongly with increased CD63-mCherry signals (Fig. 6E). In contrast, when CD63-mCherry with C→A mutations within the CCG motif (CD63_C145A,C146A_-mCherry) was used, a random distribution of fluorescence signals on the cell surface was observed (Fig. 6E). Interestingly, expression of mutated CD63-mCherry significantly reduced the recruitment of HIV-1 Gag-iGFP formations to the cell–cell contacts. Therefore, it can be assumed that endogenously expressed CD63 is displaced from the VS site because of overexpressed CD63-mCherry, which is accumulating at the cell surface and therefore cannot play an active role during cell–cell transfer. These observations taken together highly indicate that the LEL of CD63 is essential for establishing and sustaining the VS. Currently, it remains a significant challenge to explain how CD63 binds in detail to gp41. We used expressed gp41 in a post-fusion state without gp120 as well with gp120. The interaction was found predominantly at the VS when gp120 expression was driven by the HIV Gag-iGFP molecular clone and this implies that this interaction needs particular conformation of gp41 otherwise we would detect PLA signals not only exclusive at the VS. This observation indicates that gp41 interacts at a post-fusion conformation with an accessible ectodomain to CD63LEL. When we utilized HEK293T cells that are CD4 negative we do not observe such high agglomeration of PLA signals at cell–cell contacts (Fig. 5D). As the current model for cell-to-cell transfer describes interaction of Env with CD4 at the VS this would explain that the gp41 ectodomain is accessible for the LEL of CD63 when using Jurkat cells for PLA interaction experiments. In contrast, when gp120 was not expressed we were able to detect PLA signals randomized on the cell surface (Fig. 4B image B5). After identification of the CD63 domain interacting with p41 we analyzed where und under which circumstances the this PPI takes place under physiological conditions. We observed an impact of CD63 cysteine mutations within the conserved CCG motif at the VS. Further studies should determine the consequences using LEL cysteine mutations within the CCG motif of CD63 for cell-to-cell-transfer of HIV-1 particles. We assume that CD63 with its LEL binds laterally to gp41 in order to circumvent the induction of Env-mediated fusion between infected and uninfected cells. This assumption is based on the report that CD63 and CD9 block HIV-1-induced cell–cell fusion (syncytia) at the transition from hemifusion to pore opening^16^. It was also demonstrated that the formation of syncytia was repressed by overexpression of CD63 and CD9^51^. During infection, the formation of syncytia is not supportive for a sustained infection, because fused T cells will end up, after 48–72 h, in an apoptotic process^52,53^. This might indicate that HIV-1 has evolved strategies to inhibit the formation of multinucleated cells in order to survive in an infected host. We suppose that gp41 reduces its fusogenic property^54^ by binding to the LEL of CD63. This model of co-operativity between CD63 and gp41 at the VS can be strengthened by additional findings, for example by the fact that when CD63 was overexpressed in a cell–cell infection assay the cell–cell transfer of HIV-1 was reduced. Reduced cell–cell transmission was also observed when CD9 was overexpressed, but not in the case of CD81 expression^15^. Binding of the transmembrane envelope protein gp41 to CD63 at the VS would mean that the viral particles are transmitted in a controlled way to acceptor cells. Experiments analyzing the detailed mechanisms how HIV-1 utilizes CD63 in order to inhibit syncytia formation will be extremely interesting and undoubtedly important to understand the viral pathogenicity and cell–cell transfer of the virus more precisely.

In summary, the domain of interaction with gp41 was mapped to the LEL domain of CD63. Using the PLA technique, it was shown that CD63 and gp41 predominantly interact at the VS. We further found that the large extracellular loop is important for establishing the virological synapse. The findings provide novel insights concerning the mechanism how CD63 might act as a regulatory protein for cell-to-cell transfer.

## Material and methods

### Plasmids, molecular cloning and site directed mutagenesis

For the construction of prey plasmids containing a set of different CD63 domains (the amino acid [AA] sequence length is given in the Supplementary Table 1), we used the vectors pPR3-N and pPR3-SUC (Dualsystems Biotech AG, Schlieren, Switzerland). The indicated primers (Supplementary Table 2) with variable *Sfi*l restriction sites^29^ at the 5′ and 3′ regions for directional cloning were used to amplify CD63 sequence from the template vector pPR3-N-CD63^29^ by using 1 unit of PfuUltra HF polymerase (Agilent Technologies, Böblingen, Germany). The amplicons were digested with the *Sfi*I restriction enzyme (New England Biolabs, Frankfurt, Germany) overnight at 50 °C. The digested and purified fragments were ligated with T4 DNA ligase (New England Biolabs, Frankfurt, Germany) into the *Sfi*I digested vector pPR3-N or pPR3-SUC (Dualsystems Biotech AG). To generate bait plasmids pPR3-SUC-TM4LEL containing different C→A point mutation sites within the LEL of CD63, we used site directed mutagenesis. PCR reaction of a total volume of 50 μl contained 5–50 ng of the plasmid pPR3-SUC-TM4LEL, 50–125 μM of each mutation introducing primer (Supplementary Table 2), 0.25 mM dNTP mix and 2.5 units of PfuUltra HF polymerase (Agilent Technologies, Böblingen, Germany). The PCR cycles were initiated at 94 °C for 2 min, followed by 18 amplification cycles. Each PCR cycle was performed at 94 °C for 0.5 min, and the annealing temperature was set 5 °C below the T_m_ of the primers for 0.5 min, 72 °C for 1 min/kb. The PCR cycles were finished with an extension step at 72 °C for 4 min. The PCR products were treated with 20 units of *Dpn*I at 37 °C for 3 h. PCR reaction was then transformed into chemocompetent *E. coli* strain XL1-Blue (Stratagene, Heidelberg, Germany). The expression construct pCMV-CD63-mCherry was generated by using PfuUltra HF polymerase and the primers mCherry_for/mCherry_rev for amplification of the mCherry sequence from the vector template pmCherry-N1 (Clontech, Heidelberg, Germany). The amplified fragment and vector pCMV2B-CD63-FLAG^40^ were digested with *Xho*I and *Apa*I enzymes (New England Biolabs, Frankfurt, Germany), then purified and ligated with T4 DNA ligase (New England Biolabs). In the same way as described above, we introduced C→A point mutation sites in the vector pCMV-CD63-mCherry (pCMV-CD63-mCherry) and generated the vector pCMV-CD63_C145A,C146A_-mCherry. The control vector pCMV-CD63ΔLEL-mCherry was generated by cloning parts of the CD63 sequence into the vector pCMVtag2B in two steps. First, the transmembrane domain (TM) 1–3 of CD63 was amplified from the template vector pPR3-N-CD63^40^ using the primers CD63_TM1-3_for/CD63_TM1-3_for (aa 1–110 GenBank accession no. KF998086) and introduced into the vector pCMV-Tag2B through the restriction sites *BamH*I/*Pst*I; the sequence TM4 of CD63 (aa 201–238, GenBank accession no. KF998086) was amplified with the primers CD63_TM4_for/CD63_TM4_rev and introduced using the restriction sites *EcoR*V/*Hind*III. The mCherry reporter sequence was subcloned from the vector pCMV-CD63-mCherry and ligated using the restriction sites *Xho*I/*Apa*I in the vector pCMV-CD63ΔLEL. In the constructed vector pCMV-CD63ΔLEL, the mCherry tag was introduced by ligation of *Xho*I and *Apa*I digested mCherry fragment from the donor vector pCMV-CD63-mCherry. The vector pCMV-CD63_C145A,C146A_-YFP used for FRET experiments was generated by exchange of the mCherry sequence with the YFP sequence from the vector pCMV-CD63-YFP-FLAG^40^ using *Xho*I and *Apa*I restriction sites, all other vectors used were previously described^40,55^. All sequences of cloning sites were verified by Sanger sequencing.

### Yeast-based split ubiquitin interaction assay

All yeast experiments (yeast transformation, bait functionality control assay and yeast growth assay) were performed as previously described^29^. The strength of PPI was determined by yeast growth and quantification of β-galactosidase. For this, several yeast colonies transformed with bait and prey were collected and grown to an OD_600_ of 0.8–1, and all samples were diluted to an OD_600_ of 0.6; 10 µl were dropped on selective plates and incubated overnight at 30 °C. The chlorophenol-red β-d-galactopyranoside (CPRG) assay, in order to quantify the β-galactosidase gene activity, was done according the yeast protocols handbook published by Clontech.

### Cells culture and cell transfections

HEK293T cells were maintained in DMEM medium and Jurkat cells in RPMI medium supplemented with 2 mM l-glutamine, 10% fetal bovine serum, 100 U/ml penicillin and 100 µg/ml streptomycin at 37 °C in 5% CO_2_ humidified atmosphere. HEK293T cells were transfected with indicated plasmids using METAFECTENE Pro (Biontex, Munich, Germany) according to the manufacturer's instructions. Jurkat cells were transfected with the constructs HIV-1_JR-FL_Gag-iGFP and pCMV-CD63-mCherry or pCMV-CD63_C145A,C146A_-mCherry using the Amaxa nucleofection system (Amaxa Biosystems) as previously described^56^.

### Cell preparation for indirect immunofluorescence

Transfected HEK293T cells were grown in 6 wells, the cell culture was removed, and cells were fixed in 2% paraformaldehyde (PFA), diluted in PBS for 20 min at room temperature, washed 2 times with PBS and detached by pipetting. The cell suspension was dropped on poly-l-lysine treated slides within a hydrophobic marked circle area created by a pap pen (Sigma Aldrich), dried and permeabilized with 0.2% saponin (Carl Roth, Karlsruhe, Germany) in PBS for 30 min and blocked in 3% BSA (Carl Roth) in PBS for 2 h. Cells were then incubated with primary goat anti-FLAG (NB600–344, NovusBio, Littleton, USA) and mouse anti-V5 (MCA1360, Serotec, Düsseldorf, Germany) antibodies. Bound primary antibodies were visualized by incubation with fluorescently conjugated secondary antibodies for green channel anti-goat Dylight 488 (Abcam, Cambridge, UK) or red channel anti-mouse Dylight 594 (Abcam). Transfected Jurkat cells were transferred from 6 wells into a reaction tube, washed one time and fixed in 2% PFA for 20 min. Further steps were proceeded, as for the HEK293T cells. Endogenous CD63 expression was detected by incubation with mouse anti-CD63 primary antibodies (1:1 mixture of MEM259, ab8219 and NK1/C3, ab1318, Abcam), and bound primary antibodies were visualized by incubation with anti-mouse Dylight 594 (Abcam). All immunostaining procedures were performed by using 5 µg/ml of primary and 1 µg/ml of secondary antibodies diluted in 5% BSA.

### Proximity ligation assay (PLA)

Transfected HEK293T or Jurkat cells were prepared for PLA analysis as previously described^40,55^. We used the following antibodies for PLA experiments: mouse anti-V5 (MCA1360, Serotec), anti-FLAG (NB600–344, NovusBio) and anti-Na^+^, K^+^-ATPase (ab98787, Abcam). For direct PLA experiments on cells, we used the Duolink In Situ Probemaker PLUS and MINUS (Sigma Aldrich) to conjugate the antibodies, anti-2F5 (AIDS Reference and Reagent Program, National Institutes of Health, Bethesda, MD) and anti-CD63 (1:1 mixture of MEM259, ab8219 and NK1/C3, ab1318, Abcam). Jurkat cells were blocked with 5% BSA and 2% chicken albumin (Carl Roth) in PBS. The conjugated antibodies, anti-CD63 (PLA-PLUS) and anti-2F5 (PLA-MINUS), were incubated on cells at a 1:100 dilution in 5% BSA. Duolink in situ detection reagent red was used for all PLA reactions, except for Jurkat cells where detection reagent orange (Sigma Aldrich) was used. PLA samples were mounted with Duolink mounting medium (Eurogentec, Angers, France).

### Confocal laser scanning microscopy (cLSM)

All images were acquired using a Zeiss LSM 780 confocal laser scanning inverted microscope (Carl Zeiss, Oberkochen, Germany) and a 63× oil immersion objective. Fluorescence signals were detected by Zeiss ZEN smart setup instrument settings for 488 nm and 594 nm dyes. PLA images were obtained and quantification of PLA signals was performed as previously described^40,55^. Pearson correlation coefficient (PCC) was calculated using cLSM images processed by using ImageJ Fiji's plugin Coloc2 for co-localization analysis.

### FRET experiments and FRET efficiency calculation

HEK293T cells (1 × 10^5^) were seeded in an IBIDI µ-slide 8 well glass bottom. After 24 h, cells were transfected with 1 µg plasmid DNA of each indicated FRET vector using MetafectenePro (Biontex, Munich, Germany) according to the manufacturer’s instructions, and cells were incubated 24 h before FRET images were obtained. ZEN smart setup instrument settings were done for 405 nm and 514 nm dyes (multitrack channel mode, linewise switch), and the ZEN bleaching option was configured. We defined bleached ROI (red frames), unbleached ROI (green frames) and bleached background ROI (blue frames). Before starting the bleaching procedure, three pre-bleaching and three post-bleaching images were recorded. The 514 nm laser line was set at 100% laser power intensity at the bleaching time point. We calculated FRET efficiency with Zeiss ZEN FRET implemented software to obtain values for each ROI.

### Calculation of the recruiting factor *ω*

The values of the recruiting factor ω were calculated from fluorescence intensity data by using the following Eq. (1):

1 $$ \omega = \frac{{\int_{{r_{3} }}^{{r_{4} }} {I(r)dr} }}{{\int_{{r_{1} }}^{{r_{2} }} {I(r)dr} }} $$

where *I* is the fluorescence intensity, *r*_*1*_* and r*_*2*_ are intervals without cell–cell contact and *r*_*3*_ and *r*_*4*_ are intervals at the VS. FindGraph (UNIPHIZ Lab) software was used for calculation of the definite integrals.

### Western blot analysis

HEK293T cells or Jurkat cells were lysed in 100 µl 2× Laemmli buffer, chromosomal cell DNA was degraded by addition of 25 U of benzonase (Novagen, San Diego, CA); 5 µL β-mercaptoethanol was added, boiled at 95 °C for 5 min and separated by SDS-PAGE using TGX gels (BioRad, Munich, Germany); and then transferred onto an Immobilon-P PVDF membrane (Millipore, Schwalbach, Germany). Expressed proteins were probed with 0.5 µg/ml mouse anti-CD63, 1 µg/mL goat anti-FLAG, mouse anti-V5, 2 µg/mL human anti-2F5, 1 µg/mL mouse anti-α1 Na^+^,K^+^-ATPase, and 1 µg/mL of mouse anti-β actin (AM00194PU-N, Acris, Herford, Germany). Primary antibodies were detected with 0.125 µg/mL HRP-conjugated anti-mouse IgG, anti-goat IgG or anti-human IgG (DAKO, Hamburg, Germany) and visualized with Pierce ECL Western blotting substrate (Thermo Scientific, Bonn, Germany). Western blot images were acquired using a Chemocam device (Intas, Göttingen, Germany). PVDF membranes were stripped for reprobing with Roti-Free Stripping-Puffer 2.2 plus (Carl Roth).

### Statistical analysis

Results are expressed as mean ± standard deviation. A two-tailed, paired Student’s *t-*test was used to determine statistical significance (^ns^, not significant; *P < 0.05; **P < 0.01; ***P < 0.001; ****P < 0.001. A p-value < 0.05 was considered as significant between two data groups.

## Supplementary Information

Supplementary Information.
