# Supplementary material for: The large extracellular loop of CD63 interacts with gp41 of HIV-1 and is essential for establishing the virological synapse
Source: Sci Rep. 2021 May 11;11:10011. doi: 10.1038/s41598-021-89523-7 (PMC8113602; doi:10.1038/s41598-021-89523-7)
Supplement: Supplementary file 1 — Supplementary Information. [file 41598_2021_89523_MOESM1_ESM.docx]

**SUPPLEMENTARY INFORMATION**

**The Large Extracellular Loop of CD63 Interacts with gp41 of HIV-1 and is Essential for Establishing the Virological Synapse**

**Daniel Ivanusic,^1^ Kazimierz Madela,^2^ Norbert Bannert,^1^ Joachim Denner^3, 4^**

^1^HIV and Other Retroviruses, Robert Koch Institute, Nordufer 20, 13353 Berlin, Germany

^2^Special Light and Electron Microscopy, Robert Koch Institute, Nordufer 20, 13353 Berlin, Germany

^3^Robert Koch Fellow, Robert Koch Institute, Nordufer 20, 13353 Berlin, Germany

^4^present address: Institute of Virology, Department of Veterinary Medicine, Free University Berlin, 14163 Berlin, Germany

Corresponding author: Joachim.Denner@fu-berlin.de

**Supplementary Table S1:** Used primers for vector construction containing CD63 sequences, deduced amino acid (AA) sequence of the full-length CD63 protein refers to Genbank accession no. KF998086.

| **Vector** | **AA** | **Primer forward (for) reverse (rev)** |
| --- | --- | --- |
| pPR3-N-TM1-2 | 1-84 | TM1-2_CD63_for TM1-2_CD63_rev |
| pPR3-N-TM3-4 | 74-238 | TM3-4_LEL_CD63_for TM3-4_LEL_CD63_rev |
| pPR3-SUC-TM2 | 50-84 | TM2_CD63_for TM2_SEL_SIL_CD63_rev |
| pPR3-SUC-TM2SEL | 36-84 | TM2_SEL_SIL_CD63_for TM2_SEL_SIL_CD63_rev |
| pPR3-SUC-TM4 | 207-238 | TM4_CD63_for  TM2_SEL_SIL_CD63_rev |
| pPR3-SUC-TM4LEL | 108-238 | TM4_LEL_SIR_CD63_for  TM4_LEL_SIR_CD63_rev |

**Supplementary Table S2:** Sequences of primers used for molecular cloning steps.

| **Primer** | **Sequence 5´🡪 3´** |
| --- | --- |
| TM1-2_CD63_for | TTAATTAAGGCCATTACGGCCATGGCGGTGGAAGGAGGAAT |
| TM1-2_CD63_rev | TTAATTAAGGCCGAGGCGGCCAGATCATAAGACAATAGTTC |
| TM3-4_LEL_CD63_for | TTAATTAAGGCCATTACGGCCGGGGCCTGCAAGGAGAACTA |
| TM3-4_LEL_CD63_rev | TTAATTAAGGCCGAGGCGGCCACATCACCTCGTAGCCACTT |
| TM2_CD63_for | TTTTTTTGGCCATTACGGCCGGCTCTCTGTTGCCAG |
| TM2_SEL_SIL_CD63_for | TTTTTTTGGCCATTACGGCCCAGCTTGTCCTGAGTCAGACCA |
| TM2_SEL_SIL_CD63_rev | TTTTTTTGGCCGAGGCGGCCTTGATCATAAGACAATAGTTCT |
| TM4_CD63_for | TTTTTTTGGCCATTACGGCCGCTGCAGCAGCCCTTGGAA |
| TM4_LEL_SIR_CD63_for | TTTTTTTGGCCATTACGGCCAGAGATAAGGTGATGTCAGAGT |
| TM4_LEL_SIR_CD63_rev | TTTTTTTGGCCGAGGCGGCCTTCATCACCTCGTAGCCACTTC |
| C145A,C146A_CD63_LEL_for | GCAGGCAGATTTTAAGGCTGCTGGGGCTGCTAACTACACAGATTG |
| C145A,C146A _CD63_LEL_rev | CAATCTGTGTAGTTAGCAGCCCCAGCAGCCTTAAAATCTGCCTGC |
| C169A, C170A _CD63_LEL_for | GAACCGAGTCCCCGACTCCGCTGCTATTGATGTTACTGTGGG |
| C169A, C170A _CD63_LEL_rev | CCCACAGTAACATCAATAGCAGCGGAGTCGGGGACTCGGTTC |
| C177A _CD63_LEL_for | GCATTGATGTTACTGTGGGCGCTGGGATTAATTTCAACG |
| C177A _CD63_LEL_rev | CGTTGAAATTAATCCCAGCGCCCACAGTAACATCAATGC |
| C191A _CD63_LEL_for | GGCGATCCATAAGGAGGGCGCTGTGGAGAAGATTGGGGG |
| C191A _CD63_LEL_rev | CCCCCAATCTTCTCCACAGCGCCCTCCTTATGGATCGCC |
| mCherry_for | TTTTTCTCGAGATGGTGAGCAAGGGCGAGG |
| mCherry_rev | TTTTTGGGCCCATCTTGTACAGCTCGTCCATG |
| CD63_TM1-3_for | TTTTTTGGATCCATGGCGGTGGAAGGAGGAATG |
| CD63_TM1-3_rev | TTTTTCTGCAGCTTATCTCTAAACACA |
| CD63_TM4_for | TTTTTGATATCGGAGGAGGAGGAAAAAATGTGCTGG |
| CD63_TM4_rev | TTTTTTAAGCTTCATCACCTCGTAGCCACTTCT |

**Supplementary Fig. S1:** Western Blot images in full-length used for figure 4D.


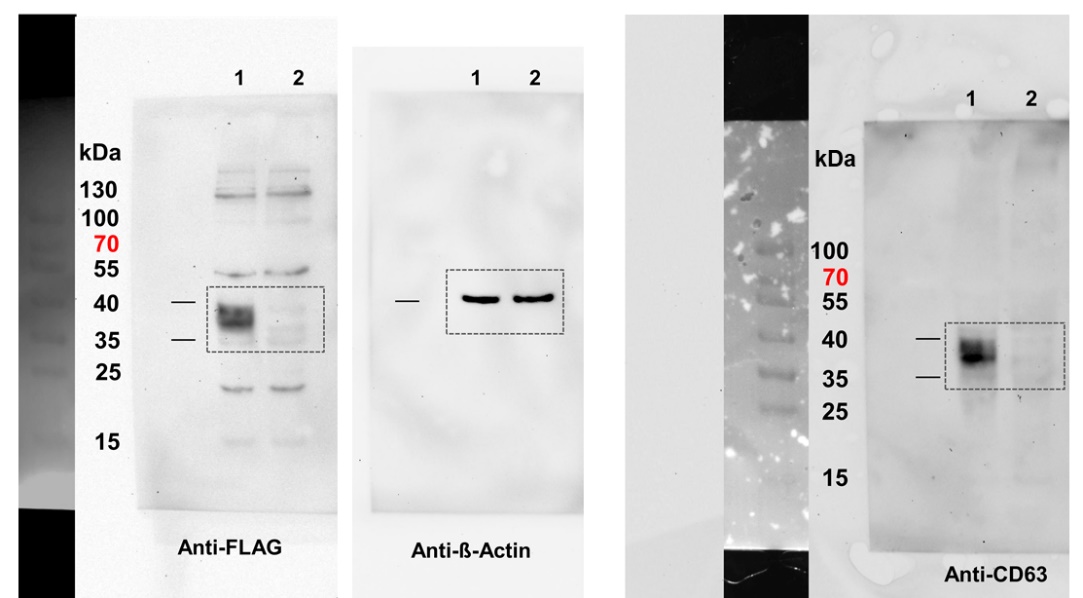


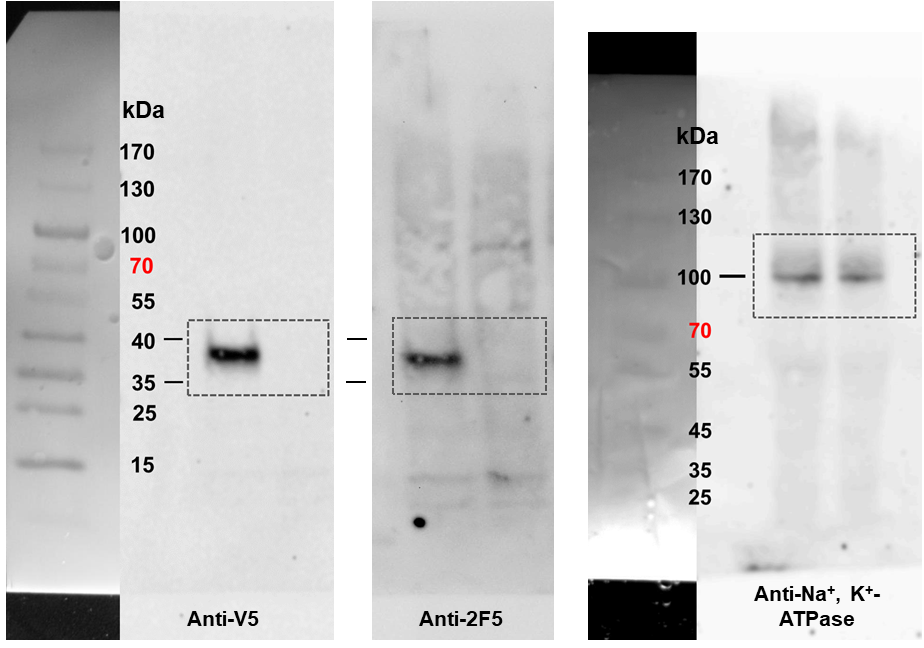


Lysates of HEK 293T cells co-transfected with the vectors lane 1: pCMV-CD63-FLAG/pcDNA-SP1-gp41-V5, lane 2: pCMV-Tag 2B/pcDNA4B

**Supplementary Fig. S2:** Western Blot images in full-length used for figure 6B.


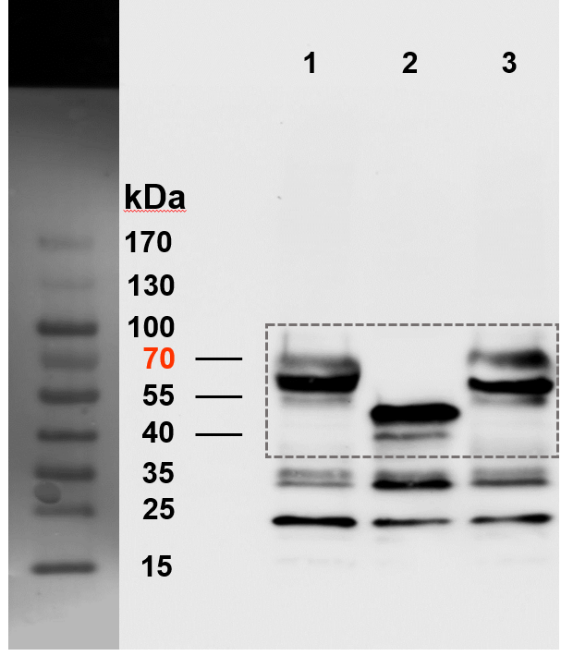


Lysates of HEK 293T cells transfected with the vectors lane1: pCMV-CD63-mCherry, lane 2: pCMV-CD63ΔLEL-mCherry, lane 3: pCMV-CD63_C145A,C146A_-mCherry.
